# Sleep and Maladaptive Personality Traits: A Twin Study

**DOI:** 10.1111/jsr.70242

**Published:** 2025-11-14

**Authors:** Ziyu Ren, Jarrod M. Ellingson, Christian Hopfer, Robert F. Krueger, Zlatan Krizan, Kristian E. Markon, Deniz S. Ones, Zoë Panchal, J. Megan Ross, Scott Vrieze, Stephanie M. Zellers, Matt McGue

**Affiliations:** ^1^ Department of Psychology University of Minnesota—Twin Cities Minneapolis Minnesota USA; ^2^ Department of Psychiatry University of Colorado Anschutz Medical Campus Aurora Colorado USA; ^3^ Institute for Behavioral Genetics University of Colorado Boulder Boulder Colorado USA; ^4^ Department of Psychology Iowa State University Ames Iowa USA; ^5^ Institute for Molecular Medicine Finland University of Helsinki Helsinki Finland

**Keywords:** AMPD, genetic correlation, maladaptive personality traits, sleep, sleep quality, twin study

## Abstract

Sleep is essential for human functioning, but little attention has been paid to the interplay between sleep and maladaptive personality traits. We examined genetic and environmental associations between sleep and four maladaptive domains (MDs)—Negative Affectivity, Detachment, Disinhibition, and Psychoticism—within the framework of the DSM‐5 Alternative Model of Personality Disorders (AMPD). We estimated phenotypic, genetic, and environmental variance and covariance using a twin study approach. Phenotypically, the four MDs showed small associations with sleep duration, medium associations with sleep efficiency, and large associations with sleep quality. A similar pattern was observed in genetic correlations, with Negative Affectivity and Detachment consistently showing the strongest associations. Multilevel Regression results based on the co‐twin control design indicated that genetic factors only partially account for these relations. Whilst previous research has extensively focused on adaptive personality traits predicting sleep, our study highlights the importance of maladaptive traits as meaningful predictors of sleep health.

## 1 Introduction

Sleep plays a crucial role in human functioning (Grandner 2022). Decades of research have linked sleep deprivation to cardiovascular health (Cincin et al. 2015) and anxiety and depression (Alvaro et al. 2013; Riemann et al. 2001). To improve sleep, it is important to understand the psychological factors that influence it. Meta‐analyses have investigated the relations between normative personality traits and sleep (Ren et al. 2025, under review). However, less attention has been paid to the relations between maladaptive personality and sleep, despite their clear relevance (Oltmanns 2019). For example, sleep problems, including insomnia, are diagnostic criteria of various mental disorders (American Psychiatric Association 2013), and have been associated with severe psychopathology characteristic of personality pathology domains (Bagautdinova et al. 2023). Therefore, we aim to explore associations between sleep and maladaptive personality domains.

The 5th edition of the *Diagnostic and Statistical Manual of Mental Disorders* (*DSM‐5*) defined personality disorders as individuals' enduring patterns of inner experiences and behaviours that markedly deviate from their cultural expectations (American Psychiatric Association 2013). The DSM‐5 Alternative Model of Personality Disorders (AMPD) delineates two major criteria. Criterion A represents the severity of personality dysfunction, capturing impairments in self‐ and interpersonal functioning (Zimmermann et al. 2020). Criterion B delineates a structural model of maladaptive personality traits (Clark and Watson 2022; Markon et al. 2024). The AMPD has received tremendous attention since its publication; it has been widely applied to explore associations between maladaptive personality traits and various clinical constructs, including spitefulness and childhood maltreatment (Rodriguez‐Seijas et al. 2019). However, only recently has research begun to examine the relations between sleep and maladaptive domains (MDs) of personality within the AMPD framework (Zakiei et al. 2025), despite early calls for relevant research (Oltmanns 2019). In addition, these studies focused on sleep disorders (e.g., insomnia) rather than normative sleep (e.g., sleep duration). To fill this gap, we used data from two population‐based twin cohorts (see Methods) to examine the relations between sleep and MDs, as well as the genetic and environmental contributions to their relations.

## 2 Maladaptive Domains of Personality and Their Associations With Sleep

Our first aim is to examine the correlations between MDs and sleep. We organised MDs under the AMPD Criterion B, which contains five domains and 25 facet traits. The domains are: Negative Affectivity, depicting frequent experiences of negative emotions and their manifestations; Detachment, portraying avoidance of socioemotional experiences; Antagonism, involving behaviours that put the individual at odds with others; Disinhibition, representing impulsive behaviours and preference for immediate gratification; and Psychoticism, describing culturally unusual behaviours and beliefs (American Psychiatric Association 2013, 789–781).

Associations between the MDs and sleep are expected, given their shared links with the widely applied Five‐Factor Model (FFM; McCrae and Costa 1997) of personality. Specifically, Clark and Watson (2022) mapped four of the MDs (i.e., all except for Psychoticism) onto the FFM using the median correlations across six samples: Antagonism to low Agreeableness, Disinhibition to low Conscientiousness, Detachment to low Extraversion, and Negative Affectivity to high Neuroticism. The only exception was Psychoticism, which did not show sizable correlations with any Big Five trait. These mapping patterns were further supported by genetic correlations (Wright et al. 2017). Overall, for four out of the five traits, the AMPD can be viewed as the maladaptive extremes of the FFM (Krueger and Markon 2014; Widiger and McCabe 2020).

Ren et al. (2025, under review) meta‐analytically examined correlations between sleep and FFM. They found that sleep duration showed trivial correlations with all FFM. Sleep continuity—the amount and distribution of sleep versus wakefulness—showed correlations around 0.30 with Neuroticism, Extraversion, and Conscientiousness. Finally, overall sleep quality, capturing participants' general experience during the night, showed correlations from 0.20 to 0.50 with Neuroticism, Extraversion, and Conscientiousness. Mechanisms linking the FFM to sleep can be extended to MDs based on their shared psychological implications. For example, moderate correlations between Neuroticism and sleep may be due to the impact of particularly high levels of negative affect and depression on sleep quality. Consequently, we expect that the MD of Negative Affectivity, which represents the extreme of Neuroticism, will show similar patterns of associations with sleep. In addition, Extraversion has also been linked to sleep (Krizan et al. 2021), leading to the expectation that the MD of Detachment, which can be conceptualised as the extreme of (low) Extraversion, will be similarly associated. Finally, the association between Conscientiousness and sleep has been attributed to the tendency of conscientious individuals to maintain consistent sleep schedules and practise good sleep hygiene (Križan and Hisler 2019), leading us to predict that the MD of Disinhibition, characterised by impulsivity and risk‐taking, will be linked to unstable sleep patterns and poor sleep quality.

In summary, research relating the FFM with sleep leads us to predict a parallel pattern of associations with MDs. We anticipated that each MD would be negatively correlated with positive sleep qualities and that the magnitude of these correlations would be trivial for sleep duration, modest for sleep continuity, and moderate for sleep quality. In addition, we expected the strongest correlations to be observed for the Negative Affectivity and Detachment domains.

## 3 Genetic and Environmental Contributions to the Maladaptive Domains—Sleep Association

Our second aim is to examine the mechanisms underlying the associations between MDs and sleep with biometric analysis. It involves decomposing the variance in a specific phenotype into portions attributable to additive genetic (A, the effect of individual genes added together), non‐additive genetic (D, the effect of interactions between alleles), shared‐environmental (C, the effect of environmental factors shared by individuals reared together), and non‐shared environmental (E, the effect of environmental factors that are not shared by individuals reared together) factors. These factors are typically combined into models including ACE, ADE, and AE where AE, for example, signifies a model that includes only additive genetic (A) and non‐shared environmental (E) factors. The proportion of phenotypic variance associated with additive genetic effects is usually called the heritability and designated as *a* ^2^.

In a sample of US adult twins, fitting AE models, Wright et al. (2017) reported evidence that all five MDs were moderately heritable with no evidence for shared environmental effects. Similarly, for sleep, Kocevska et al. (2021) concluded that a substantial portion of individual differences in sleep was due to genetic effects. Given the heritability of both MDs and sleep, the observed correlation between them can be either causal (i.e., higher levels of MDs lead to more sleep problems or conversely sleep disturbance contributes to MDs risk) or due to genetic confounding (i.e., the association is not causal but rather due to overlapping genetic effects, or pleiotropy; Solovieff et al. 2013), or both. We addressed this question using the cotwin control design (McGue et al. 2010). Specifically, associations within genetically identical monozygotic (MZ) pairs eliminate the possibility of genetic (as well as shared environmental) confounding, whilst associations within genetically non‐identical dizygotic (DZ) pairs control for 50% of shared genetic effects (and shared environmental effects). If an association were due to genetic confounding, we would consequently expect that the effect would be eliminated within MZ twins and reduced but not eliminated within DZ pairs. Alternatively, if there is a causal association, then control for genetic confounding would not eliminate the association. In our case, the absence of the association between MDs and sleep within MZ twins would indicate that the relationship is likely due to shared genetic influences. Alternatively, if within‐pair effects remain significant within MZ twins, it would suggest potential causal associations.

## 4 The Current Study

Given the expected links between MDs and sleep, we aimed to fill the gaps in the literature by (1) establishing the correlations between MDs and sleep and (2) exploring the extent to which these correlations can be explained by genetic or shared environmental confounding. To achieve these goals, in a sample of more than 2000 twins, we (1) estimated their phenotypic correlations, (2) conducted univariate and bivariate biometric analyses to estimate their heritability and genetic and environmental contributions to their covariance, and (3) utilized the cotwin control design to assess the degree of genetic or shared environmental confounding in the observed relations. We then leveraged these findings towards informing developmental connections between maladaptive personality traits and sleep health.

## 5 Materials and Methods

### 5.1 Participants

The current study used anonymized data drawn from two twin cohorts: the Colorado Twin Registry (Corley et al. 2019) and the Minnesota Twin Family Study (Iacono and McGue 2002). Both cohorts employed a longitudinal approach, with twin cohorts initially ascertained through public records (birth records in Minnesota and school records in Colorado), first assessed when the twins were in adolescence, and followed longitudinally through early adulthood. The current study used sleep and AMPD data that were collected remotely from 2018 to 2021, when the twins were in early adulthood. We removed participants who lacked either demographic information (*N* = 3), sleep records (*N* = 233), or AMPD scores (*N* = 58). We also removed one outlier who reported it took 630 min to fall asleep. The resulting sample included 2802 participants, with 980 twin pairs where both members participated (641 MZ pairs and 339 same‐sex DZ pairs). At the time of assessment, twins had an average age of 35.37 years (SD = 5.37, range = 24.80 to 49.20) and the majority were female (61.10%).

### 5.2 Procedure

Assessments at both the Colorado and Minnesota sites were undertaken remotely. Twins who had participated in previous longitudinal research were contacted by phone or email, given a description of the project, and invited to voluntarily participate. Those who agreed completed an online Qualtrics survey, which took about 1 h to complete, and a telephone assessment, which also took about 1 h. The study was approved by the IRBs at both the University of Minnesota and the University of Colorado and participants received a $100 honorarium for completing their assessment. The sleep and personality disorders data used in the current study came from self‐report Qualtrics surveys. Due to constraints on time available for all the project's assessments, only 13 of the 25 AMPD traits were administered. They covered all five MDs except Antagonism.

### 5.3 Measures and Scoring

#### 5.3.1 Demographics

For demographic data, sex (male = 1, female = 2), zygosity (MZ = 1, DZ = 2), race, and ethnicity were self‐reported. Participants reported their race as White, Black, Native American, Asian, Native Hawaiian or Pacific Islander, mixed race or multiracial, or another race, and their ethnicity as Hispanic or non‐Hispanic. Age was calculated from the participants' date of birth and date of survey response. Table 1 shows the demographic data separately for the Colorado and Minnesota sites. Comparison between the Colorado and Minnesota samples did not show statistically significant differences in sample characteristics; therefore, data from these two sites were combined in the analysis and were reported together in later results.

**TABLE 1:** Demographics of Colorado and Minnesota sites.

Factor	Total sample	Minnesota	Colorado
*N*	2802	1616	1186
Age			
Mean	35.37	36.39	33.98
SD	5.37	6.52	2.64
Range	24.8–49.2	24.8–49.2	27.9–41.7
Gender			
%Male	38.90%	39.09%	37.52%
% Female	61.10%	60.09%	62.48%
Race			
% White	92.90%	96.47%	88.03%
% Black	0.32%	0.00%	0.76%
% American Indian	0.89%	0.80%	1.01%
% Asian	1.07%	0.62%	1.69%
% Pacific Islander	0.78%	1.18%	0.25%
% Multi‐racial	3.32%	0.93%	6.58%
% Unknown	0.71%	0.00%	1.69%
Ethnicity			
%Hispanic	4.53%	1.30%	8.94%
%Non‐hispanic	95.46%	98.70%	91.06%

#### 5.3.2 Sleep

Sleep variables were measured using the 20‐item Pittsburgh Sleep Quality Inventory (PSQI; Buysse et al. 1989). The PSQI assesses sleep duration and sleep efficiency on a continuous scale, sleep duration in hours, and sleep efficiency as a percentage of total time asleep divided by total time in bed. The remaining domains—sleep latency (time taken to fall asleep), sleep disturbance (difficulty falling asleep), sleep medication (usage of medication to aid sleep), daytime dysfunction (ability to stay energetic during the day), and subjective sleep quality (self‐rated overall sleep quality) – were measured on Likert scales ranging from 1 (e.g., Not during the past month) to 4 (e.g., Three or more times a week), and their scores were calculated following PSQI's manual.

We conducted factor analysis to group the seven domains into more homogeneous categories. An exploratory factor analysis (EFA) with oblimin rotation identified three factors, supported by parallel analysis. The three factors were: (1) sleep duration, with only sleep duration as the prominent loading item (> 0.60); (2) sleep efficiency, which loaded principally on the sleep efficiency and sleep latency scales; and (3) sleep quality, which loaded on all the remaining sleep components. The rotated loadings are given in Table S1 and factor intercorrelations are given in Table S2. To compute sleep scores for each factor, the sleep duration item score was retained as the sleep duration score; the sleep efficiency item score was kept as the sleep efficiency score (sleep latency was dropped because it contains similar content to sleep efficiency); and the scores for sleep disturbance, sleep medication, daytime dysfunction, and subjective sleep quality were summed and then reverse coded as the sleep quality score. For all scores, higher scores indicate better sleep.

#### 5.3.3 AMPD Traits

Personality disorders were assessed for 13 of the 25 AMPD facets. Each facet was assessed with 4 items using the abbreviated PID‐5 scales derived by Maples et al. (2015). Each item was rated on a 0 (Very False or Often False) to 3 (Very True or Often True) scale and aggregated into facet scores following the rules specified by Maples et al. Following Krueger et al. (2012), four AMPD domain scores were calculated by averaging the AMPD facet scores: (1) Negative Affectivity with facets Anxiousness and Restricted Affectivity (α = 0.65); (2) Detachment with facets Anhedonia, Depressivity, Intimacy Avoidance, and Withdrawal (α = 0.81); (3) Disinhibition with facets Risk Taking, Impulsivity, Distractibility, and Irresponsibility (α = 0.73); and (4) Psychoticism with facets Eccentricity, Cognitive and Perceptual Dysregulation, and Unusual Beliefs & Experiences (α = 0.71). Although meta‐analytic evidence suggests Restricted Affectivity may better reflect Detachment (Watters and Bagby 2018), in our data, it shows stronger correlations with Anxiousness than Detachment facets (See Table S3), consistent with the model upon which the PID‐5 is based. Therefore, we followed the original AMPD scoring and categorised it under Negative Affectivity. To further explore the influence of a general personality pathology factor (*p*‐Factor; Zimmermann et al. 2020), estimated the *p*‐Factor with the mean of the 13 facets (see Table S4 and its accompanying explanation for the rationale behind this approach). The *p*‐Factor may also serve as a proxy for Criterion A severity.

### 5.4 Data Analysis Procedure

All data analyses were conducted using R (R Core Team 2025). Amongst the 2802 participants, 2 individuals were missing scores on some MD domains (0.07%), and sleep latency scores were missing for 324 individuals (11.57%). Missing data were imputed using the k‐Nearest Neighbours approach with *k* = 5 (Mucherino et al. 2009). For all variables, data were winsorized to z‐scores between −3 and 3.

To answer our first question, we computed the phenotypic correlations between the age‐sex corrected AMPD traits and sleep variables. We adjusted for the linear effects of age and sex by taking the residuals from regressing each sleep variable on age and sex (McGue and Bouchard 1984).

To examine the genetic contributions to the relations between sleep and AMPD traits, we conducted biometric analyses with the “OpenMx” package (Boker et al. 2025). For univariate models, we fitted ACE, AE, and ADE models for the three sleep variables and four AMPD traits and evaluated the overall fit using Akaike's Information Criterion (AIC; Bozdogan 1987), with a modified script from the OpenMx documentation (https://openmx.ssri.psu.edu/docs/OpenMx/2.1.0/GeneticEpi_Matrix.html#ace‐model‐a‐twin‐analysis). This was followed by bivariate biometric analyses, where we fitted ACE, AE, and ADE models for the 12 sleep and AMPD pairs and evaluated their fit using AIC. We used a script modified from Dr. Hermine Maes' website of bivariate models estimating path coefficients (https://hermine‐maes.squarespace.com/#/two/). All R codes for the current analysis can be found on the online Open Science Framework (https://osf.io/zydjm/overview?view_only=be442e797c294797807eb6c778391024).

Finally, we examined the extent to which familial confounding impacts the MD and sleep relations. To achieve this, we used a mixed‐level regression framework (Begg and Parides 2003; Gurrin et al. 2006; McGue et al. 2010) to estimate within‐ and between‐pair effects of AMPD traits on sleep. We denoted $y_{\mathrm{ij}}$ as the observed sleep outcome for the *j*th twin (*j* = 1, 2) in the *i*th twin pair (*i* = 1, 2, …, *N*), $x_{\mathrm{ij}}$ as the observed AMPD score, ${\bar{x}}_{i.}$ the mean observed AMPD score in the *i*th twin pair. The model without an interaction effect (i.e., unpooled effects) was:

(1) $$y_{\mathrm{ij}}=\beta_{0}+\beta_{W}x_{\mathrm{ij}}+\beta_{B}{\bar{x}}_{i.}+\varepsilon_{\mathrm{ij}}$$

where $\beta_{0}$ represents the intercept, $\beta_{W}$ the within‐pair effect, $\beta_{B}$ the between‐pair effect. The regression model with a zygosity interaction effect was:

(2) $$y_{\mathrm{ij}}=\beta_{0}+\beta_{W}x_{\mathrm{ij}}+\beta_{B}{\bar{x}}_{i.}+\beta_{I}x_{\mathrm{ij}}\times \text{Zygosity}+\varepsilon_{\mathrm{ij}}$$

where $\beta_{I}$ represents the interacting effect of zygosity. We calculated and reported the pooled effect sizes for MZ and DZ twins, separately. These two models were fitted for all 12 pairs of sleep and AMPD using the “lme4” package (Bates et al. 2015).

## 6 Results

Descriptive statistics for sleep and MDs, together with those for their lower order facets, are shown in Table 2. Phenotypic correlations and their 95% Confidence Intervals (CIs), controlled for age and sex, are reported in Table 3. Correlations without controls and between lower‐order sleep and MD facets can be found in the Tables S3 and S5–S7. Sleep factors showed moderate inter‐correlations (range from 0.11 to 0.36); MDs displayed moderate to strong inter‐correlations (range from 0.49 to 0.63). MDs' correlations were generally small with sleep duration (−0.08 to −0.10); medium with sleep efficiency (−0.14 to −0.23); and large to very large with Sleep Quality (−0.28 to −0.42; Funder and Ozer 2019). Whilst non‐trivial correlations with sleep were observed with all MDs, the strongest correlations were consistently seen with Negative Affectivity and Detachment. In addition, the *p*‐Factor showed a similar pattern of correlations, with magnitudes that are comparable to those of Detachment: small with sleep duration (−0.09), medium with efficiency (−0.24), and strong with quality (−0.44). Overall, these observations supported our hypothesis that sleep and MDs would be negatively correlated, with patterns that parallel those observed between sleep and FFM.

**TABLE 2:** Descriptive data of sleep domains and maladaptive domain (MD) traits and facets.

Variable	Mean	SD	Skewness
Sleep duration	7.89	1.09	−0.10
Sleep efficiency	0.96	0.04	−2.13
Sleep quality	8.78 (Sleep quality is reverse‐coded)	1.91	−1.02
Sleep disturbance	1.16	0.49	0.37
Sleep medication	0.47	0.96	1.88
Daytime dysfunction	0.49	0.66	1.01
Subjective sleep quality	1.10	0.69	0.47
MD negative affectivity	3.85	2.45	0.49
Anxiousness	3.75	3.02	0.52
Reverse‐coded (lack of) restricted affectivity	3.95	2.66	0.64
MD detachment	1.70	1.74	1.08
Anhedonia	1.84	2.21	1.15
Depressivity	0.82	1.59	2.03
Intimacy avoidance	1.47	2.16	1.54
Withdrawal	2.65	2.66	0.71
MD disinhibition	1.75	1.46	0.84
Risk taking	1.49	1.71	1.09
Impulsivity	1.73	1.80	0.97
Distractibility	3.40	3.13	0.63
Irresponsibility	0.37	0.88	2.66
MD psychoticism	1.27	1.49	1.24
Eccentricity	2.38	2.75	0.98
Cognitive and perceptual dysregulation	0.39	0.84	2.15
Unusual beliefs & experiences	1.04	1.66	1.65
*p*‐factor (mean of facets)	1.93	1.44	0.72

**TABLE 3:** Phenotypic correlations between sleep and maladaptive domains (MDs) controlled for age and sex.

Variable	1. [CI]	2. [CI]	3. [CI]	4. [CI]	5. [CI]	6. [CI]	
1. Sleep duration							
2. Sleep efficiency	0.32 [0.29, 0.35]						
3. Sleep quality	0.12 [0.09, 0.16]	0.36 [0.33, 0.39]					
4. MD negative affectivity	−0.08 [−0.12, −0.05]	−0.20 [−0.24, −0.16]	−0.37 [−0.40, −0.34]				
5. MD detachment	−0.07 [−0.11, −0.04]	−0.23 [−0.27, −0.20]	−0.42 [−0.45, −0.39]	0.57 [0.55, 0.60]			
6. MD psychoticism	−0.10 [−0.13, −0.06]	−0.17 [−0.21, −0.13]	−0.28 [−0.31, −0.25]	0.49 [0.46, 0.52]	0.54 [0.51, 0.56]		
7. MD disinhibition	−0.06 [−0.10, −0.03]	−0.14 [−0.17, −0.10]	−0.33 [−0.37, −0.30]	0.50 [0.48, 0.53]	0.55 [0.53, 0.58]	0.63 [0.61, 0.65]	
8. *p*‐factor	−0.09 [−0.13, −0.06]	−0.23 [−0.26, −0.19]	−0.44 [−0.47, −0.41]	0.78 [0.77, 0.79]	0.85 [0.84, 0.86]	0.79 [0.79, 0.80]	0.84 [0.83, 0.85]

Abbreviation: CI = 95% confidence interval.

MZ and DZ twin correlations and results of univariate biometric analysis based on best‐fitting AE models are shown in Table 4 (Since previous studies on the heritability of both AMPD (Wright et al. 2017) and sleep (Kocevska et al. 2021) supported the AE model, we only reported results of AE model in the current paper, and results for univariate and bivariate ACE and ADE models in the Supporting Information) (ACE and ADE results were included in the Table S8). Our results supported heritability for all three sleep factors, with the weakest heritability estimate for sleep duration (*a* ^2^ = 0.17). For MDs, *a* ^2^ ranged from 0.34 to 0.43. Consistent with previous studies (Kocevska et al. 2021; Wright et al. 2017), non‐shared environmental factors (E) explained more variance compared to additive genetic factors (A).

**TABLE 4:** Univariate AE estimates of sleep and maladaptive domains (MDs).

Variable	Observed correlations		Biometric parameters	
	*r*_MZ_ [CI]	*r*_DZ_ [CI]	*a* ^2^ [CI]	*e* ^2^ [CI]
1. Sleep duration	0.20 [0.12, 0.27]	−0.04 [−0.15, −0.06]	0.17 [0.15, 0.24]	0.83 [0.76, 0.85]
2. Sleep efficiency	0.33 [0.25, 0.39]	0.17 [0.07, 0.27]	0.32 [0.25, 0.38]	0.68 [0.63, 0.75]
3. Sleep quality	0.32 [0.24, 0.38]	0.02 [−0.09, 0.13]	0.30 [0.23, 0.36]	0.70 [0.64, 0.77]
4. MD negative affectivity	0.34 [0.27, 0.41]	0.21 [0.11, 0.31]	0.34 [0.28, 0.40]	0.66 [0.60, 0.72]
5. MD detachment	0.43 [0.36, 0.49]	0.12 [0.01, 0.22]	0.41 [0.34, 0.46]	0.59 [0.54, 0.66]
6. MD psychoticism	0.44 [0.37, 0.50]	0.18 [0.08, 0.28]	0.44 [0.38, 0.50]	0.56 [0.50, 0.62]
7. MD disinhibition	0.43 [0.36, 0.49]	0.19 [0.08, 0.29]	0.43 [0.37, 0.49]	0.57 [0.51, 0.63]
8. *p*‐factor	0.46 [0.39, 0.52]	0.20 [0.09, 0.30]	0.46 [0.40, 0.51]	0.54 [0.48, 0.60]

Abbreviations: AE = models with addictive genetic (A) and non‐shared environmental (E) effects; CI = 95% confidence interval; *r*_DZ_ = observed correlations between (arbitrarily assigned) dizygotic twin1 and twin2, *N* = 339 pairs; *r*_MZ_ = observed correlations between (arbitrarily assigned) monozygotic twin1 and twin2, *N* = 641 pairs.

Results for bivariate AE models are shown in Table 5 (ACE and ADE results were included in the Tables S9–S10). For MDs, we observed small to medium genetic correlations (*r*_a_) with sleep duration (between −0.04 and −0.19), medium to large genetic correlations with sleep efficiency (between −0.28 and −0.45), and very large genetic correlations with sleep quality (between −0.49 and −0.67). Large genetic correlations were consistently seen with the Negative Affectivity and Detachment dimensions. Non‐shared environmental correlations (*r*_e_) were small for sleep duration (between −0.06 and −0.08) and sleep efficiency (between −0.06 and −0.16), and medium for sleep quality (between −0.17 and −0.29). Bivariate heritability estimates (i.e., the proportion of the phenotypic correlation attributable to overlapping genetic effects) were strong for both sleep efficiency (between 0.58 and 0.76) and sleep quality (between 0.55 and 0.63), and moderate for sleep duration (between 0.16 and 0.48). A similar pattern of results was observed for the *p*‐factor, with slightly higher magnitudes. In summary, a moderate to large portion of phenotypic correlations between MD and sleep factors was based on their shared genetic factors.

**TABLE 5:** Bivariate AE estimates of sleep and maladaptive domains (MDs).

		MD negative affectivity	MD detachment	MD psychoticism	MD disinhibition	*p*‐factor
Sleep duration	*r*_a_ [CI]	−0.19 [−0.41, 0.02]	−0.12 [−0.33, 0.07]	−0.12 [−0.32, 0.07]	−0.04 [−0.24, 0.15]	−0.12 [−0.31, 0.06]
	*r*_e_ [CI]	−0.07 [−0.13, 0.00]	−0.06 [−0.13, 0.01]	−0.08 [−0.16, −0.02]	−0.08 [−0.15, −0.01]	−0.10 [−0.17, −0.02]
	*r*_p_	−0.09	−0.07	−0.09	−0.07	−0.10
	[CI]	[−0.13, −0.05]	[−0.11, −0.03]	[−0.13, −0.05]	[−0.11, −0.03]	[−0.14, −0.06]
	*h* ^2^	0.48	0.43	0.36	0.16	0.35
Sleep efficiency	*r*_a_ [CI]	−0.45 [−0.59, −0.31]	−0.39 [−0.52, −0.26]	−0.34 [−0.47, −0.21]	−0.28 [−0.41, −0.15]	−0.42 [−0.54, −0.30]
	*r*_e_ [CI]	−0.08 [−0.15, −0.01]	−0.16 [−0.23, −0.09]	−0.06 [−0.14, 0.00]	−0.06 [−0.13, 0.01]	−0.13 [−0.19, −0.05]
	*r*_p_	−0.20	−0.24	−0.17	−0.14	−0.24
	[CI]	[−0.24, −0.16]	[−0.28, −0.20]	[−0.21, −0.13]	[−0.18, −0.10]	[−0.28, −0.20]
	*h* ^2^	0.71	0.56	0.74	0.72	0.66
Sleep quality	*r*_a_ [CI]	−0.65 [−0.79, −0.52]	−0.67 [−0.79, −0.55]	−0.49 [−0.62, −0.36]	−0.56 [−0.69, −0.43]	−0.69 [−0.80, −0.58]
	*r*_e_ [CI]	−0.25 [−0.31, −0.18]	−0.29 [−0.35, −0.22]	−0.17 [−0.24, −0.10]	−0.22 [−0.29, −0.15]	−0.30 [−0.37, −0.24]
	*r*_p_	−0.37	−0.42	−0.28	−0.34	−0.44
	[CI]	[−0.41, −0.34]	[−0.45, −0.38]	[−0.32, −0.24]	[−0.37, −0.30]	[−0.48, −0.41]
	*h* ^2^	0.56	0.56	0.63	0.59	0.58

Abbreviations: AE = models with addictive genetic (A) and non‐shared environmental (E) effects; CI = 95% Confidence Intervals; *h* ^2^ = bivariate heritability estimate; *r*_e_ = environmental correlation; *r*_e_ = phenotypical correlation; *r*_g_ = genetic correlation.

To understand the impact of genetic confounds in the associations between MDs and sleep, we fitted mixed‐level regression models as described in Equations (1) and (2), using MDs to predict sleep. This analysis is best seen as being complementary to the bivariate biometric analyses in that it quantifies effects in terms of the magnitude of within‐pair regression coefficients rather than as genetic and non‐shared environmental correlations. Table 6 shows the effect sizes and their corresponding 95% CIs for predicting sleep duration, sleep efficiency, and sleep quality, respectively. For models without a zygosity interaction, we report estimates pooled across zygosity (Pooled effect sizes for MZ and DZ twins were estimated by fitting mixed‐level regression models on MZ and DZ data, separately). In models testing zygosity interactions, most interaction terms were nonsignificant—except when Detachment and Psychoticism predicted Sleep Duration. Full interaction effects are available in Tables S11–S13. Although we report separate within‐pair effects for MZs and DZs, we focus on the pooled results in our interpretation because of the limited evidence that the effects varied by zygosity. Note that a finding that a within‐pair effect did not vary by zygosity is consistent with the association not being attributable entirely to genetic confounding.

**TABLE 6:** Mixed‐level regression results of using maladaptive traits (with and without interaction effect with Zygosity) to predict sleep outcomes.

Sleep	MD	Pooled effects		Unpooled effects		M‐*R* ^2^	C‐*R* ^2^
		$\beta_{W}$ [95% CI]	$\beta_{B}$ [95% CI]	$\beta_{W-\mathrm{MZ}}$ [95% CI]	$\beta_{W-\mathrm{DZ}}$ [95% CI]		
Sleep duration	MD negative affectivity	−0.07* [−0.14, 0.00]	−0.05 [−0.14, 0.04]	−0.10* [−0.19, −0.02]	−0.02 [−0.13, 0.10]	0.01	0.12
	MD detachment	−0.05 [−0.12, 0.02]	−0.04 [−0.13, 0.05]	−0.13** [−0.22, −0.04]	0.04 [−0.07, 0.15]	0.01	0.12
	MD psychoticism	−0.09* [−0.16, −0.01]	0.00 [−0.09, 0.09]	−0.13** [−0.22, −0.04]	−0.03 [−0.15, 0.09]	0.01	0.12
	MD disinhibition	−0.07 [−0.15, 0.00]	0.00 [−0.10, 0.09]	−0.15** [−0.24, −0.06]	0.03 [−0.09, 0.15]	0.01	0.12
	*p*‐factor	−0.09* [−0.16, −0.01]	−0.02 [−0.12, 0.07]	−0.18*** [−0.27, −0.08]	0.02 [−0.08, 0.08]	0.01	0.12
Sleep efficiency	MD negative affectivity	−0.09** [−0.15, −0.03]	−0.17*** [−0.26, −0.08]	−0.09* [−0.17, −0.01]	−0.09 [−0.19, 0.02]	0.05	0.28
	MD detachment	−0.18*** [−0.24, −0.12]	−0.10* [−0.18, −0.01]	−0.19*** [−0.27, −0.10]	−0.17*** [−0.27, −0.08]	0.06	0.30
	MD psychoticism	−0.09** [−0.15, −0.02]	−0.12** [−0.21, −0.04]	−0.08 [−0.17, 0.00]	−0.09 [−0.20, 0.01]	0.03	0.28
	MD disinhibition	−0.06 [−0.12, 0.01]	−0.12** [−0.21, −0.03]	−0.09* [−0.17, 0.00]	−0.02 [−0.12, 0.09]	0.02	0.28
	*p*‐factor	−0.14*** [−0.21, −0.08]	−0.14*** [−0.22, −0.05]	−0.16*** [−0.25, −0.07]	−0.12* [−0.23, −0.02]	0.06	0.29
Sleep quality	MD negative affectivity	−0.29*** [−0.35, −0.22]	−0.13** [−0.22, −0.05]	−0.27*** [−0.34, −0.19]	−0.32*** [−0.43, −0.21]	0.15	0.28
	MD detachment	−0.36*** [−0.42, −0.29]	−0.10* [−0.18, −0.02]	−0.31*** [−0.39, −0.23]	−0.41*** [−0.51, −0.31]	0.18	0.30
	MD psychoticism	−0.23*** [−0.30, −0.17]	−0.08 [−0.17, 0.01]	−0.18*** [−0.26, −0.09]	−0.30*** [−0.42, −0.19]	0.08	0.25
	MD disinhibition	−0.28*** [−0.34, −0.21]	−0.10* [−0.19, −0.02]	−0.26*** [−0.34, −0.18]	−0.30*** [−0.41, −0.18]	0.12	0.26
	*p*‐factor	−0.39*** [−0.45, −0.32]	−0.09* [−0.17, 0.00]	−0.36*** [−0.44, −0.27]	−0.43*** [−0.53, −0.32]	0.20	0.31

Abbreviations: $\beta_{B}$ = between‐pair effect; $\beta_{W}$ = within‐pair effect; $\beta_{W-\mathrm{DZ}}$ = DZ within‐pair effect including interactions; $\beta_{W-\mathrm{MZ}}$ = MZ within‐pair effect including interactions; CI = confidence interval; MD = maladaptive domains; C‐*R* ^2^ = conditional *R* ^2^; M‐*R* ^2^ = marginal *R* ^2^; pooled effect sizes = results from models with NO interaction; unpooled effect sizes = results from models with interaction.

* *p* < 0.01.

** *p* < 0.001.

When using MDs to predict sleep duration, the pooled within‐pair effects were small and only significant for Psychoticism and Negative Affectivity. The finding of MZ effects indicates that the association cannot be attributed entirely to genetic confounding. When predicting sleep efficiency, modest and significant within‐pair effects were found for all MDs except for Disinhibition ($\beta_{W}$ ranging from −0.09 to −0.18). In general, within‐pairs, higher level of MDs was associated with worse sleep efficiency. In addition, modest and significant between‐pair effects were found for all four MDs ($\beta_{B}$ ranging from −0.10 to −0.17), indicating that twin pairs with higher level of AMPDs tend to have worse sleep efficiency. Since the interaction effects were negligible for most models, we concluded that genetic factors only have a small confounding effect. When predicting sleep quality, moderate to large significant within‐pair effects were found for all MDs traits with medium magnitudes ($\beta_{W}$ ranging from −0.23 to −0.36); higher MDs predicted worse sleep quality. Modest significant between‐pair effects were found for Negative Affectivity and Disinhibition. Genetic factors only partially explained the association. Similar patterns of results were observed for *p*‐Factor, with slightly higher within‐pair effects when predicting sleep quality, though with highly overlapped CIs with Detachment. Finally, small improvements were found from conditional *R* ^2^ values to marginal *R* ^2^, supporting the importance of considering pair‐level effects.

## 7 Discussion

We explored the phenotypic and genetic associations between sleep and MDs. Extending meta‐analysis findings on the FFM (Ren et al. 2025, under review), our results supported the hypotheses that MDs are negatively correlated with sleep, both phenotypically and genetically. In addition, all sleep and MD variables were moderately heritable. However, genetic factors only partially confounded their relations, providing evidence consistent with—but not conclusively establishing—a causal association.

The three aspects of sleep showed distinct patterns of correlations with MDs. Sleep duration showed the smallest correlations with MDs; people with more severe MDs tend to sleep less. Similarly, sleep efficiency exhibited small to medium negative correlations with MDs; people with a higher level of MDs tend to take longer to fall asleep. This may be due to their elevated anxiety levels, physiological arousal, or increased rumination before sleep (Krizan et al. 2024). Finally, sleep quality showed medium to very large phenotypic correlations with MDs. These patterns were also observed in the *p*‐Factor. This may be attributed to three main reasons. First, having a severe MD may lead to a worse sleep experience. For example, people high in Negative Affectivity may ruminate more before sleep, and people high in Disinhibition may maintain inconsistent sleep schedules and poor sleep hygiene (Križan and Hisler 2019). Second, MDs may not only contribute to objectively poorer sleep quality but also predispose people to perceive and report their sleep more negatively. Third, disrupted sleep may precipitate or maintain affective or self‐regulatory dysfunction, which in turn may aggravate PD symptomatology over time (Hisler et al. 2019).

Replicating previous findings, our univariate genetic results supported the heritability of both sleep (Kocevska et al. 2021) and MDs (Wright et al. 2017). Results from bivariate biometric analyses extended the literature by demonstrating the genetic correlations between them. Specifically, for sleep duration and MDs, we observed considerable non‐shared environmental effects, with bivariate AE models showing small to moderate genetic and environmental correlations. This result may reflect pervasive and unpredictable constraints on sleep timing that are exogenous to the individual (work schedules, family obligations). Regarding sleep efficiency, moderate to strong genetic correlations were found for all four MDs, whereas small to moderate non‐shared environmental correlations were only seen for Negative Affectivity and Detachment. Finally, strong genetic correlations were found between all four MDs and sleep quality. In general, our findings showed that common genetic factors have a large influence on the correlations between MDs and sleep. Although the *p*‐factor did not demonstrate stronger associations with sleep than MDs, its largely comparable and sometimes slightly higher correlations suggest that it may serve as a meaningful summary indicator linking personality pathology to sleep.

Using a cotwin control design, mixed‐level regression consistently showed that genetic factors partially confounded the associations between MDs and all three sleep variables. However, after having controlled for interactions with zygosity, significant within‐pair effects persist amongst MZ twins. These results are consistent with the causal hypothesis that either more severe MDs lead to worse sleep outcomes or conversely that poor sleep contributes to MDs. Whilst the causal pathways by which MDs might affect sleep are discussed above, the pathway from poorer sleep to more severe MDs also deserves attention. For example, disrupted sleep can enhance emotional reactivity (Altena et al. 2016), which may in turn increase irritability and impulsivity. Over time, these changes could lead to worse MDs.

These results are also clinically important, as the bidirectional causal pathways suggest the importance of evaluating for both sleep and MDs when either is the presenting concern in a clinical encounter. It is also consistent with evidence that cognitive behavioural therapy for insomnia is more effective than behavioural interventions alone; that is, addressing negative cognitions and affect is important for effectively improving sleep for individuals with insomnia (Edinger et al. 2021). In addition, whilst our data did not support a general pathology factor, future studies using a more comprehensive assessment of all PID‐5 facets could further investigate this issue. The results may inform whether clinical treatment should focus on generalised emotional regulation, or more targeted approaches, such as reducing anxiety related to Negative Affectivity (We thank an anonymous reviewer for raising the importance of considering shared variance across MDs and its clinical implications, which prompted additional analyses and discussion of the general factor).

## 8 Limitations and Future Directions

The current study has several limitations. First, the data included only 13 out of the 25 maladaptive facets and only four of five MDs. Our assessment did not investigate the impact of Antagonism, which research on FFM Agreeableness suggests might be negatively associated with sleep quality (Ren et al. 2025; under review). Furthermore, we did not include some major facets that load strongly on the Negative Affectivity domain, such as Emotional Liability and Depressivity (Clark and Watson 2022; Krueger et al. 2012). These two facets may have strong negative correlations with sleep due to their associations with more mood swings and negative experiences. In addition, although we included Restricted Affectivity as part of Negative Affectivity, prior research has questioned this classification (Watters and Bagby 2018). Therefore, our assessment provides only a limited representation of the Negative Affectivity domain. This limited coverage of facets also restricted our ability to robustly measure the *p*‐Factor and to extract unique variance from individual MDs. Second, our sample primarily consisted of middle‐aged twins. Previous meta‐analyses have shown that sleep patterns vary across the lifespan and can shift throughout development (Ohayon et al. 2004), so the results may change for samples of other age groups. Finally, our sample was predominantly White and female, which may limit the generalizability of the findings to more diverse or clinical populations.

Future studies could also extend the current finding by measuring sleep through objective measures. PSQI results may be influenced by subjective response bias; people experiencing higher Negative Affectivity may report a longer time to fall asleep and more complaints about their sleep quality. Objective measures, such as actigraphy, are easy to implement and are not subject to such biases, though they have their own limitations. By comparing objective sleep data with subjective sleep ratings and their associations with MDs, we can better understand the extent to which these covariates are due to response patterns (Križan and Hisler 2019). For example, previous studies using actigraphy to assess sleep duration in adulthood have reported higher heritability estimates (0.45; Kocevska et al. 2021), whereas our self‐reported sleep duration yielded a lower estimate (0.17). Incorporating objective measures in future research would help better account for measurement bias.

## 9 Conclusions

The current study is the first to examine associations between sleep and MDs under the framework of AMPD. Phenotypically, we found that sleep duration, sleep efficiency, and sleep quality are all negatively correlated with MDs. Genetically, these associations are only partially explained by shared genetic factors. Notably, sleep quality showed very large phenotypic and genetic correlations with MDs, and Detachment and Negative Affectivity appeared to be the two most influential traits correlated with sleep. Future research should continue to explore these associations to deepen our understanding of the complex interplay between MDs and sleep.

## Author Contributions

**Ziyu Ren:** conceptualization, methodology, software, investigation, validation, formal analysis, visualization, writing – original draft, writing – review and editing. **Jarrod M. Ellingson:** conceptualization, data curation, funding acquisition, writing – review and editing. **Christian Hopfer:** conceptualization, funding acquisition, writing – review and editing. **Robert F. Krueger:** conceptualization, writing – review and editing. **Zlatan Krizan:** conceptualization, writing – review and editing. **Kristian E. Markon:** conceptualization, writing – review and editing. **Deniz S. Ones:** supervision, writing – original draft, writing – review and editing. **Zoë Panchal:** writing – review and editing. **J. Megan Ross:** conceptualization, writing – review and editing. **Scott Vrieze:** conceptualization, data curation, funding acquisition. **Stephanie M. Zellers:** writing – review and editing. **Matt McGue:** conceptualization, methodology, data curation, investigation, validation, supervision, funding acquisition, visualization, project administration, resources, writing – original draft, writing – review and editing. All authors reviewed and approved the final version of the manuscript.

## Ethics Statement

This study is based on previously collected, de‐identified data. The original data collection procedure was approved by the IRBs at both the University of Minnesota and the University of Colorado.

## Conflicts of Interest

The authors declare no conflicts of interest.

## Supporting information


**Table S1:** Factor Pattern Matrix for EFA with Oblimin Factor Rotation on PSQI with Three‐Factor Solution.
**Table S2:** Factor Correlation Matrix for EFA with Oblimin Factor Rotation on PSQI with Three‐Factor Solution.
**Table S3:** Observed Correlations Between AMPD Facets.
**Table S4:** Factor Loadings and Fit Statistics for PID‐5 Bi‐Factor Model.
**Table S5:** Observed Correlations amongst Sleep and MDs.
**Table S6:** Observed Correlations Between PSQI Domains.
**Table S7:** Observed Correlations Between AMPD Facets and PSQI Domains.
**Table S8:** Univariate ACE and ADE Estimates of Sleep and Maladaptive Domains.
**Table S9:** Bivariate ACE Estimates of Sleep and Maladaptive Domains.
**Table S10:** Bivariate ADE Estimates of Sleep and Maladaptive Domains.
**Table S11:** Mixed‐level Regression Results of using AMPD (with and without interaction effect with Zygosity) to Predict Sleep Duration.
**Table S12:** Mixed‐level Regression Results of using AMPD (with and without interaction effect with Zygosity) to Predict Sleep Efficiency.
**Table S13:** Mixed‐level Regression Results of using AMPD (with and without interaction effect with Zygosity) to Predict Sleep Quality.

## Data Availability

Access to subject data is limited due to restrictions put in place by the Colorado Multiple Institutional Review Board (COMIRB). The IRB will not permit release of these subjects‐data to a public archive as participants were not asked to consent to such release and during the consent process subjects placed limitations on the ways their study data could be used. Qualified researchers may apply for data access at https://ibg.colorado.edu/dokuwiki/doku.php?id=ibg_data:start. Data requests will be overseen by the Principle Investigator as stated by the COMIRB (comirb@ucdenver.edu).
