# Supplementary material for: Sleep and Maladaptive Personality Traits: A Twin Study
Source: J Sleep Res. 2025 Nov 14;35(3):e70242. doi: 10.1111/jsr.70242 (PMC13193377; doi:10.1111/jsr.70242)
Supplement: Supplementary file 1 — Table S1: Factor Pattern Matrix for EFA with Oblimin Factor Rotation on PSQI with Three‐Factor Solution. Table S2: Factor Correlation Matrix for EFA with Oblimin Factor Rotation on PSQI with Three‐Factor Solution. Table S3: Observed Correlations Between AMPD Facets. Table S4: Factor Loadings and Fit Statistics for PID‐5 Bi‐Factor Model. Table S5: Observed Correlations amongst Sleep and MDs. Table S6: Observed Correlations Between PSQI Domains. Table S7: Observed Correlations Between AMPD Facets and PSQI Domains. Table S8: Univariate ACE and ADE Estimates of Sleep and Maladaptive Domains. Table S9: Bivariate ACE Estimates of Sleep and Maladaptive Domains. Table S10: Bivariate ADE Estimates of Sleep and Maladaptive Domains. Table S11: Mixed‐level Regression Results of using AMPD (with and without interaction effect with Zygosity) to Predict Sleep Duration. Table S12: Mixed‐level Regression Results of using AMPD (with and without interaction effect with Zygosity) to Predict Sleep Efficiency. Table S13: Mixed‐level Regression Results of using AMPD (with and without interaction effect with Zygosity) to Predict Sleep Quality. [file JSR-35-e70242-s001.docx]

**Table S1.**

*Factor Pattern Matrix for EFA with Oblimin Factor Rotation on PSQI with Three-Factor Solution .*

| **PSQI Components** | **F1. Sleep Duration** | **F2. Sleep Efficiency** | **F3. Sleep Quality** |
| --- | --- | --- | --- |
| **1. Sleep Duration** | **0.68** | 0.02 | 0.04 |
| **2. Sleep Latency** | -0.10 | **0.82** | 0.10 |
| **3. Sleep Efficiency** | 0.10 | **0.86** | -0.07 |
| **4. Subjective Sleep Quality** | 0.17 | 0.11 | **0.58** |
| **5. Sleep Disturbance** | -0.10 | 0.12 | **0.49** |
| **6. Daytime Dysfunction** | 0.00 | -0.09 | **0.65** |
| **7. Sleep Medication** | -0.12 | 0.15 | **0.30** |

*Note: EFA = Exploratory Factor Analysis; PSQI = Pittsburgh Sleep Quality Index; for each component, the highest factor loading is bolded. RMSAD = 0.008 for the current model solution, indicating an excellent fit.*

**Table S2.**

*Factor Correlation Matrix for EFA with Oblimin Factor Rotation on PSQI with Three-Factor Solution .*

|  | **F1. Sleep Duration** | **F2. Sleep Efficiency** | **F3. Sleep Quality** |
| --- | --- | --- | --- |
| **F1. Sleep Duration** | 1.00 |  |  |
| **F2. Sleep Efficiency** | 0.22 | 1.00 |  |
| **F3. Sleep Quality** | 0.10 | 0.48 | 1.00 |

*Note: EFA = Exploratory Factor Analysis; PSQI = Pittsburgh Sleep Quality Index.*

**Table S3.**

*Observed Correlations Between AMPD Facets.*

|  | **1.** | **2.** | **3.** | **4.** | **5.** | **6.** | **7.** | **8.** | **9.** | **10.** | **11.** | **12.** | **13.** |
| --- | --- | --- | --- | --- | --- | --- | --- | --- | --- | --- | --- | --- | --- |
| **1. Anxiousness** | 1.00 |  |  |  |  |  |  |  |  |  |  |  |  |
| **2. (lack of) Restricted Affectivity** | -0.49 | 1.00 |  |  |  |  |  |  |  |  |  |  |  |
| **3. Anhedonia** | 0.53 | -0.36 | 1.00 |  |  |  |  |  |  |  |  |  |  |
| **4. Depressivity** | 0.44 | -0.28 | 0.77 | 1.00 |  |  |  |  |  |  |  |  |  |
| **5. Intimacy Avoidance** | 0.29 | -0.27 | 0.45 | 0.40 | 1.00 |  |  |  |  |  |  |  |  |
| **6. Withdrawal** | 0.48 | -0.40 | 0.59 | 0.51 | 0.50 | 1.00 |  |  |  |  |  |  |  |
| **7. Risk Taking** | 0.11 | -0.25 | 0.25 | 0.24 | 0.17 | 0.23 | 1.00 |  |  |  |  |  |  |
| **8. Impulsivity** | 0.30 | -0.26 | 0.40 | 0.36 | 0.24 | 0.31 | 0.56 | 1.00 |  |  |  |  |  |
| **9. Distractibility** | 0.55 | -0.36 | 0.50 | 0.43 | 0.32 | 0.46 | 0.30 | 0.50 | 1.00 |  |  |  |  |
| **10. Irresponsibility** | 0.30 | -0.2 | 0.42 | 0.46 | 0.28 | 0.34 | 0.38 | 0.44 | 0.41 | 1.00 |  |  |  |
| **11. Eccentricity** | 0.39 | -0.35 | 0.43 | 0.40 | 0.30 | 0.46 | 0.46 | 0.47 | 0.50 | 0.40 | 1.00 |  |  |
| **12. Cognitive and Perceptual Dysregulation** | 0.30 | -0.29 | 0.37 | 0.40 | 0.30 | 0.35 | 0.38 | 0.39 | 0.35 | 0.44 | 0.5 | 1.00 |  |
| **13. Unusual Beliefs & Experiences** | 0.35 | -0.32 | 0.35 | 0.36 | 0.28 | 0.36 | 0.37 | 0.40 | 0.37 | 0.38 | 0.54 | 0.61 | 1.00 |

*Note: AMPD = Alternative Model of Personality Disorders. (Lack of) Restricted Affectivity was reverse-coded when used to create AMPD domain scores.*

**Table S4.**

*Factor Loadings and Fit Statistics for PID-5 Bi-Factor Model.*

| Facet | **p-Factor** | **NegAff** | **Disin** | **Detach** | **Psycho** |
| --- | --- | --- | --- | --- | --- |
| **1. Anxiousness** | 0.66 | 0.33 |  |  |  |
| **2. (lack of) Restricted Affectivity** | 0.50 | 0.47 |  |  |  |
| **3. Anhedonia** | 0.71 |  |  | 0.63 |  |
| **4. Depressivity** | 0.64 |  |  | 0.51 |  |
| **5. Intimacy Avoidance** | 0.49 |  |  | 0.17 |  |
| **6. Withdrawal** | 0.67 |  |  | 0.18 |  |
| **7. Risk Taking** | 0.42 |  | 0.53 |  |  |
| **8. Impulsivity** | 0.57 |  | 0.62 |  |  |
| **9. Distractibility** | 0.72 |  | 0.11 |  |  |
| **10. Irresponsibility** | 0.65 |  | 0.23 |  |  |
| **11. Eccentricity** | 0.67 |  |  |  | 0.27 |
| **12. Cognitive and Perceptual Dysregulation** | 0.56 |  |  |  | 0.48 |
| **13. Unusual Beliefs & Experiences** | 0.57 |  |  |  | 0.61 |

*Note: Model fit indices are χ²(46) = 16443.311,* p *< .001; CFI = .93; TLI = .89; RMSEA = .091, 90% CI [.09, .10], SRMR = .05.*

**Interpretation.** In response to a reviewer’s request, we fit a bi-factor model to the PID-5 data. The general factor from this model shows that all facets contribute relatively equally, and the general factor score correlated very strongly (r = .87) with a simple mean score across the facets. On this basis, we treated the mean score as an adequate representation of the general p-factor. In contrast, the unique factors are difficult to interpret. Because we did not administer all PID-5 facet scales, there is a limited number of facets available under each dimension and the factor loadings for these unique factors appear unstable. In general, researchers look for H statistics of .70 or higher (Rodriguez et al., 2016). In our case, the H statistics are as follow: 0.89 for p-Factor, 0.52 for Disinhibition, 0.51 for Detachment, 0.49 for Psychoticism, and 0.29 for Negative Affectivity. In addition, while the model yielded acceptable fit indices, there’s debate on the applicability of the bifactor models to psychopathology (e.g., Watts et al., 2019). Importantly, as reported in the main tables, the general factor score showed negative correlations with sleep outcomes in a pattern consistent with the main text—weak for sleep duration, moderate for sleep efficiency, and strong for sleep quality—providing convergent support for our primary findings.

**Supplementary References**

Rodriguez, A., Reise, S. P., & Haviland, M. G. (2016). Evaluating bifactor models: Calculating and interpreting statistical indices. *Psychological Methods*, 21(2), 137–150. <https://doi.org/10.1037/met0000045>

Watts, A. L., Poore, H. E., & Waldman, I. D. (2019). Riskier tests of the validity of the bifactor model of psychopathology. *Clinical Psychological Science, 7*(6), 1285–1303. <https://doi.org/10.1177/2167702619855035>

**Table S5.**

*Observed Correlations among Sleep and MDs.*

| **Variable** | **1.** | **2.** | **3.** | **4.** | **5.** | **6.** | **7.** |
| --- | --- | --- | --- | --- | --- | --- | --- |
| **1. Sleep Duration** |  |  |  |  |  |  |  |
| **2. Sleep Efficiency** | 0.31 |  |  |  |  |  |  |
| **3. Sleep Quality** | 0.11 | 0.36 |  |  |  |  |  |
| **4. Negative Affectivity** | -0.07 | -0.21 | -0.38 |  |  |  |  |
| **5. Detachment** | -0.08 | -0.23 | -0.41 | 0.56 |  |  |  |
| **6. Psychoticism** | -0.11 | -0.16 | -0.27 | 0.47 | 0.54 |  |  |
| **7. Disinhibition** | -0.08 | -0.13 | -0.32 | 0.49 | 0.56 | 0.65 |  |
| **8. p-Factor** | -0.10 | -0.22 | -0.43 | 0.76 | 0.85 | 0.79 | 0.84 |

*Note: Sleep duration is measured in hours, sleep efficiency as a proportion, and sleep quality on a 12-point Likert scale.*

**Table S6.**

*Observed Correlations Between PSQI Domains.*

|  | **1.** | **2.** | **3.** | **4.** | **5.** | **6.** | **7.** |
| --- | --- | --- | --- | --- | --- | --- | --- |
| **1. Sleep Duration** | 1.00 |  |  |  |  |  |  |
| **2. Sleep Latency** | 0.09 | 1.00 |  |  |  |  |  |
| **3. Sleep Efficiency** | 0.23 | 0.71 | 1.00 |  |  |  |  |
| **4. Subjective Sleep Quality** | 0.21 | 0.40 | 0.34 | 1.00 |  |  |  |
| **5. Sleep Disturbance** | 0.01 | 0.33 | 0.01 | 0.35 | 1.00 |  |  |
| **6. Daytime Dysfunction** | 0.06 | 0.24 | 0.06 | 0.39 | 0.31 | 1.00 |  |
| **7. Sleep Medication** | -0.02 | 0.26 | 0.21 | 0.21 | 0.23 | 0.23 | 1.00 |

*Note: PSQI = Pittsburgh Sleep Quality Index. PSQI domains 4 - 7 are reverse-coded so that higher scores indicate better sleep for all variables.*

**Table S7.**

*Observed Correlations Between AMPD Facets and PSQI Domains.*

|  | **Duration** | **Latency** | **Efficiency** | **Sub-Quality** | **Disturbance** | **Dysfunction** | **Medication** |
| --- | --- | --- | --- | --- | --- | --- | --- |
| **1. Anxiousness** | -0.01 | -0.28 | -0.21 | -0.27 | -0.26 | -0.37 | -0.21 |
| **2. (lack of) Restricted Affectivity** | 0.05 | 0.15 | 0.09 | 0.15 | 0.15 | 0.23 | 0.11 |
| **3. Anhedonia** | -0.03 | -0.25 | -0.20 | -0.30 | -0.22 | -0.47 | -0.15 |
| **4. Depressivity** | -0.06 | -0.2 | -0.17 | -0.26 | -0.19 | -0.43 | -0.13 |
| **5. Intimacy Avoidance** | -0.02 | -0.18 | -0.14 | -0.16 | -0.13 | -0.27 | -0.11 |
| **6. Withdrawal** | -0.06 | -0.18 | -0.14 | -0.19 | -0.18 | -0.33 | -0.12 |
| **7. Risk Taking** | -0.07 | -0.07 | -0.04 | -0.08 | -0.07 | -0.14 | -0.01 |
| **8. Impulsivity** | -0.06 | -0.10 | -0.07 | -0.13 | -0.14 | -0.21 | -0.06 |
| **9. Distractibility** | -0.02 | -0.20 | -0.13 | -0.22 | -0.19 | -0.41 | -0.16 |
| **10. Irresponsibility** | -0.04 | -0.15 | -0.09 | -0.17 | -0.18 | -0.32 | -0.10 |
| **11. Eccentricity** | -0.07 | -0.15 | -0.10 | -0.17 | -0.16 | -0.27 | -0.05 |
| **12. Cognitive and Perceptual Dysregulation** | -0.02 | -0.11 | -0.05 | -0.11 | -0.13 | -0.19 | -0.07 |
| **13. Unusual Beliefs & Experiences** | -0.08 | -0.14 | -0.12 | -0.18 | -0.17 | -0.23 | -0.07 |

*Note:; PSQI = Pittsburgh Sleep Quality Index; AMPD: Alternative Model of Personality Disorders. (Lack of) Restricted Affectivity was reverse-coded when used to create AMPD domain scores.*

**Table S8.**

*Univariate ACE and ADE Estimates of Sleep and Maladaptive Domains.*

|  |  |  |  |  | **ACE Model** | | |  | **ADE Model** | | | | |
| --- | --- | --- | --- | --- | --- | --- | --- | --- | --- | --- | --- | --- | --- |
| **Variable** | **Zygosity** | **r** | **N** | **a^2^_ACE_[CI]** | **c^2^_ACE_** | **e^2^_ACE_[CI]** | **AIC_ADE_** |  | **a^2^_ADE_[CI]** | **d^2^_ADE_** | **e^2^_ADE_[CI]** | **AIC_ADE_** |  |
| **1. Sleep Duration** | MZ  DZ | 0.20  -0.05 | 641  339 | 0.16  [0.10, NA] | 0.00  [NA, 0.08] | 0.83  [NA, 0.90] | 6624.31 |  | 0.00  [NA, NA] | 0.18  [0.02, NA] | 0.82  [NA, 0.89] | 6621.31 |  |
| **2. Sleep Efficiency** | MZ  DZ | 0.33  0.17 | 641  339 | 0.27  [0.04, 0.38] | 0.04  [NA, 0.25] | 0.68  [0.62, 0.75] | 6483.89 |  | 0.32  [NA, NA] | 0.00  [NA, 0.35] | 0.68  [0.62, NA] | 6484.04 |  |
| **3. Sleep Quality** | MZ  DZ | 0.32  0.02 | 641  339 | 0.30  [0.18, NA] | 0.00  [NA, 0.07] | 0.70  [NA, 0.77] | 6630.99 |  | 0.00  [NA, 0.24] | 0.32  [0.05, NA] | 0.68  [NA, 0.75] | 6626.15 |  |
| **4. Negative Affectivity** | MZ  DZ | 0.34  0.21 | 641  339 | 0.25  [0.02, NA] | 0.08  [NA, 0.28] | 0.66  [0.60, 0.73] | 6579.70 |  | 0.34  [0.28, NA] | 0.00  [NA, 0.29] | 0.66  [0.59, NA] | 6583.22 |  |
| **5. Detachment** | MZ  DZ | 0.43  0.12 | 641  339 | 0.41  [0.31, NA] | 0.00  [NA, NA] | 0.59  [NA, NA] | 6506.09 |  | 0.05  [NA, 0.43] | 0.36  [NA, NA] | 0.58  [0.52, 0.64] | 6503.15 |  |
| **6. Psychoticism** | MZ  DZ | 0.44  0.18 | 641  339 | 0.44  [0.32, NA] | 0.00  [NA, 0.10] | 0.56  [NA, NA] | 6472.97 |  | 0.22  [NA, 0.49] | 0.32  [NA, 0.51] | 0.54  [0.48, 0.61] | 6471.31 |  |
| **7. Disinhibition** | MZ  DZ | 0.43  0.19 | 641  339 | 0.43  [0.28, NA] | 0.00  [NA, NA] | 0.57  [NA, 0.62] | 6469.01 |  | 0.29  [NA, 0.48] | 0.15  [NA, NA] | 0.56  [0.50, 0.62] | 6468.50 |  |
| **8. p-Factor** | MZ  DZ | 0.46  0.19 | 641  339 | 0.46  [0.32, NA] | 0.00  [NA, NA] | 0.54  [NA, 0.59] | 1785.04 |  | 0.29  [NA, NA] | 0.18  [NA, NA] | 0.53  [0.48, 0.59] | 1784.23 |  |

*Note: ACE = models with additive genetic (A), shared environmental (C), and non-shared environmental (E) effects; ADE = models with additive genetic (A), non-additive genetic (D), and non-shared environmental (E) effects. a² = proportion of variance due to additive genetic effects; c² = shared environmental effects; d² = non-additive genetic effects; e² = non-shared environmental effects. MZ = monozygotic twins, N = 641 pairs; DZ = dizygotic twins, N = 339 pairs. CI = 95% confidence interval; AIC = Akaike Information Criterion (lower values indicate better model fit).*

**Table S9.**

*Bivariate ACE Estimates of Sleep and Maladaptive Domains.*

|  |  | **Negative Affectivity** | **Detachment** | **Psychoticism** | **Disinhibition** | **p-Factor** |
| --- | --- | --- | --- | --- | --- | --- |
| **Sleep Duration** | **r_a_**  **[CI]** | -0.05  [-0.65, 1.00] | -0.12  [-0.39, 0.24] | -0.12  [-0.44, 0.17] | -0.04  [-0.28, NA] | -0.12  [-0.37, 0.36] |
|  | **r_c_**  **[CI]** | 1.00  [1.00, 1.00] | -0.51  [-0.51, 1.00] | -0.87  [NA, 0.94] | -0.94  [NA, NA] | -0.98  [NA, 0.99] |
|  | **r_e_**  **[CI]** | -0.07  [-0.14, 0.00] | -0.06  [-0.13, 0.01] | -0.09  [-0.16, -0.02] | -0.08*  [-0.15, -0.01] | -0.10*  [-0.16, -0.02] |
|  | **AIC** | 13229.60 | 13164.55 | 13123.33 | 13127.16 | 13112.75 |
|  | **χ^2^** | 29.04* | 48.22* | 33.74* | 30.56* | 34.80* |
| **Sleep Efficiency** | **r_a_**  **[CI]** | -0.15  [-0.66, 1.00] | -0.39*  [NA, -0.03] | -0.31  [NA, 0.09] | -0.10  [-0.48, 1.00] | -0.35  [-0.72, -0.35] |
|  | **r_c_**  **[CI]** | 1.00  [1.00, 1.00] | -1.00  [-1.00, NA] | -1.00  [-1.00, NA] | -1.00  [-1.00, 0.98] | -1.00  [NA, 1.00] |
|  | **r_e_**  **[CI]** | -0.10  [-0.16, -0.03] | -0.16*  [-0.23, -0.09] | -0.07  [-0.14, 0.00] | -0.06  [-0.14, 0.00] | -0.13*  [-0.20, -0.05] |
|  | **AIC** | 13011.04 | 12901.94 | 12939.81 | 12954.45 | 12870.19 |
|  | **χ^2^** | 24.34 | 30.14* | 46.01* | 25.16 | 29.12* |
| **Sleep Quality** | **r_a_**  **[CI]** | -0.66*  [-1.00, -0.29] | -0.67*  [-0.89, -0.55] | -0.49*  [-0.79, -0.35] | -0.56*  [-0.77, -0.38] | -0.69  [-0.91, -0.58] |
|  | **r_c_**  **[CI]** | -1.00  [-1.00, 1.00] | 1.00  [1.00, 1.00] | -0.98  [NA, 1.00] | 0.16  [NA, 1.00] | 1.00  [NA, NA] |
|  | **r_e_**  **[CI]** | -0.25*  [-0.31, -0.18] | -0.29*  [-0.35, -0.22] | -0.16*  [-0.24, -0.10] | -0.22*  [-0.28, -0.15] | -0.30  [-0.37, -0.23] |
|  | **AIC** | 12920.49 | 12753.32 | 12967.09 | 12879.35 | 12661.54 |
|  | **χ^2^** | 19.41 | 32.69* | 28.16* | 21.12 | 21.06 |

*Note: ACE = models with additive genetic (A), shared environmental (C), and non-shared environmental (E) effects; r_g_ = genetic correlation; r_e_ = environmental correlation; CI = 95% Confidence Intervals;* χ^2^ *= Chi-squared value comparing to saturated model.*

** p < .05*

**Table S10.**

*Bivariate ADE Estimates of Sleep and Maladaptive Domains.*

|  |  | **Negative Affectivity** | **Detachment** | **Psychoticism** | **Disinhibition** | **p-Factor** |
| --- | --- | --- | --- | --- | --- | --- |
| **Sleep Duration** | **r_a_**  **[CI]** | -1.00  [-1.00, 1.00] | -1.00  [-1.00, 1.00] | 1.00  [-1.00, 1.00] | -1.00  [-1.00, 1.00] | -1.00  [-1.00, 1.00] |
|  | **r_d_**  **[CI]** | 1.00  [-1.00, 1.00] | -0.02  [-1.00, 1.00] | -0.19  [-1.00, 1.00] | 0.65  [-1.00, 1.00] | 0.42  [-1.00, 1.00] |
|  | **r_e_**  **[CI]** | -0.07  [-0.14, 0.00] | -0.07  [-0.14, 0.01] | -0.09  [-0.16, -0.01] | -0.10*  [-0.17, -0.02] | -0.11*  [-0.18, -0.03] |
|  | **AIC** | 13227.10 | 13158.50 | 13118.89 | 13122.50 | 13108.12 |
|  | **χ^2^** | 26.54 | 42.17* | 29.30* | 25.89 | 30.17* |
| **Sleep Efficiency** | **r_a_**  **[CI]** | -1.00  [-1.00, -0.24] | -1.00  [-1.00, 1.00] | -0.85  [-1.00, 1.00] | -1.00  [-1.00, -0.15] | -1.00  [NA, -0.99] |
|  | **r_d_**  **[CI]** | 1.00  [-1.00, 1.00] | 1.00  [-1.00, 1.00] | 1.00  [-1.00, 1.00] | -1.00  [-1.00, 1.00] | 1.00  [-0.96, NA] |
|  | **r_e_**  **[CI]** | -0.09  [-0.16, -0.02] | -0.16*  [-0.23, -0.09] | -0.07  [-0.15, 0.00] | -0.07  [-0.14, 0.00] | -0.14  [-0.21, -0.06] |
|  | **AIC** | 13012.68 | 12898.86 | 12937.65 | 12952.53 | 12867.73 |
|  | **χ^2^** | 25.99 | 27.07 | 43.84* | 23.25 | 26.66 |
| **Sleep Quality** | **r_a_**  **[CI]** | -1.00  [-1.00, 1.00] | -1.00  [-1.00, 1.00] | -1.00  [-1.00, 1.00] | -1.00  [-1.00, 1.00] | -1.00  [NA, 1.00] |
|  | **r_d_**  **[CI]** | -1.00  [-1.00, 0.48] | -0.67  [-1.00, 0.98] | -0.71  [-1.00, 0.98] | -0.45  [-1.00, 0.94] | -0.74  [-1.00, 1.00] |
|  | **r_e_**  **[CI]** | -0.25*  [-0.31, -0.18] | -0.29*  [-0.35, -0.22] | -0.16*  [-0.23, -0.08] | -0.22*  [-0.28, -0.15] | -0.30*  [-0.37, -0.23] |
|  | **AIC** | 12916.63 | 12746.66 | 12960.89 | 12874.48 | 12656.90 |
|  | **χ^2^** | 15.55 | 26.03 | 21.96 | 16.26 | 16.41 |

*Note: ADE = models with additive genetic (A), non-additive genetic (D), and non-shared environmental (E) effects; r_g_ = genetic correlation; r_e_ = environmental correlation; CI = 95% Confidence Intervals;* χ^2^ *= Chi-squared value comparing to saturated model.*

** p < .05*

**Table S11.**

*Mixed-level Regression Results of using AMPD (with and without interaction effect with Zygosity) to Predict* ***Sleep Duration****.*

| **Model** | **Model** | $\boldsymbol{\beta}_{\boldsymbol{W}}$ **(95% CI)** | $\boldsymbol{\beta}_{\boldsymbol{B}}$ **(95% CI)** | $\boldsymbol{\beta}_{\boldsymbol{I}}$ **(95% CI)** | **Marginal R^2^** | **Conditional R^2^** |
| --- | --- | --- | --- | --- | --- | --- |
| **Interaction** | **Negative Affectivity** | -0.13  [-0.27, 0.02] | -0.02  [-0.11, 0.06] | -0.02  [-0.11, 0.08] | 0.01 | 0.14 |
|  | **Detachment** | -0.26***  [-0.42, -0.11] | -0.03  [-0.12, 0.06] | 0.15**  [0.05, 0.24] | 0.01 | 0.14 |
|  | **Psychoticism** | -0.26***  [-0.41, -0.11] | 0.00  [-0.09, 0.09] | -0.12*  [0.03, 0.21] | 0.01 | 0.14 |
|  | **Disinhibition** | -0.23**  [-0.38, -0.07] | -0.01  [-0.10, 0.09] | 0.10  [0.02, 0.21] | 0.01 | 0.14 |
|  | **p-Factor** | -0.30**  [-0.45, -0.14] | 0.00  [-0.10, 0.09] | 0.15*  [0.05, 0.24] | 0.02 | 0.13 |
| **No Interaction** | **Negative Affectivity** | -0.07  [-0.14, 0.00] | 0.05  [-0.14, 0.04] | - | 0.01 | 0.12 |
|  | **Detachment** | -0.05  [-0.12, 0.02] | -0.04  [-0.13, 0.05] | - | 0.01 | 0.12 |
|  | **Psychoticism** | -0.09*  [-0.16, -0.01] | 0.00  [-0.09, 0.09] | - | 0.01 | 0.12 |
|  | **Disinhibition** | -0.07  [-0.15, 0.00] | 0.00  [-0.10, 0.08] | - | 0.01 | 0.12 |
|  | **p-Factor** | -0.09*  [-0.16, -0.01] | -0.02  [-0.12, 0.07] | - | 0.01 | 0.12 |

*Note: AMPD = Alternative Model of Personality Disorders;* $\beta_{W}$ ***=*** *within-pair effect;* $\beta_{B}$ ***=*** *between-pair effect;* $\beta_{I}$ ***=*** *Interaction effect with zygosity; CI = Confidence Interval.*

** p < 0.05; ** p < 0.01; *** p < 0.001.*

**Table S12.**

*Mixed-level Regression Results of using AMPD* *(with and without interaction effect with Zygosity) to Predict* ***Sleep Efficiency****.*

| **Model** | **Predictor** | $\boldsymbol{\beta}_{\boldsymbol{W}}$ **(95% CI)** | $\boldsymbol{\beta}_{\boldsymbol{B}}$ **(95% CI)** | $\boldsymbol{\beta}_{\boldsymbol{I}}$ **(95% CI)** | **Marginal R^2^** | **Conditional R^2^** |
| --- | --- | --- | --- | --- | --- | --- |
| **Interaction** | **Negative Affectivity** | -0.05  [-0.18, 0.09] | -0.18***  [-0.27, -0.09] | -0.03  [-0.11, 0.06] | 0.05 | 0.28 |
|  | **Detachment** | -0.22**  [-0.36, -0.08] | -0.08  [-0.17, 0.00] | 0.03  [-0.06, 0.12] | 0.06 | 0.30 |
|  | **Psychoticism** | -0.08  [-0.23, 0.06] | -0.11*  [-0.20, -0.02] | -0.01  [-0.09, 0.09] | 0.03 | 0.28 |
|  | **Disinhibition** | -0.10*  [-0.25, -0.05] | -0.10*  [-0.20, -0.02] | 0.03  [-.06, 0.12] | 0.02 | 0.28 |
|  | **p-Factor** | -0.15*  [-0.29, 0.00] | -0.14***  [-0.23, -0.05] | 0.00  [-0.09, 0.09] | 0.06 | 0.29 |
| **No Interaction** | **Negative Affectivity** | -0.09**  [-0.15, -0.03] | -0.17***  [-0.26, -0.08] | - | 0.05 | 0.28 |
|  | **Detachment** | -0.18***  [-0.24, -0.12] | -0.10*  [-0.18, -0.01] | - | 0.06 | 0.30 |
|  | **Psychoticism** | -0.09**  [-0.15, -0.02] | -0.12**  [-0.21, -0.04] | - | 0.03 | 0.28 |
|  | **Disinhibition** | -0.06  [-0.12, 0.01] | -0.12**  [-0.21, -0.03] | - | 0.02 | 0.28 |
|  | **p-Factor** | -0.14***  [-0.21, -0.08] | -0.14***  [-0.22, -0.05] | - | 0.06 | 0.29 |

*Note: AMPD = Alternative Model of Personality Disorders;* $\beta_{W}$ ***=*** *within-pair effect;* $\beta_{B}$ ***=*** *between-pair effect;* $\beta_{I}$ ***=*** *Interaction effect with zygosity; CI = Confidence Interval.*

** p < 0.05; ** p < 0.01; *** p < 0.001.*

**Table S13.**

*Mixed-level Regression Results of using AMPD (with and without interaction effect with Zygosity) to Predict* ***Sleep Quality****.*

| **Model** | **Predictor** | $\boldsymbol{\beta}_{\boldsymbol{W}}$ **(95% CI)** | $\boldsymbol{\beta}_{\boldsymbol{B}}$ **(95% CI)** | $\boldsymbol{\beta}_{\boldsymbol{I}}$ **(95% CI)** | **Marginal R^2^** | **Conditional R^2^** |
| --- | --- | --- | --- | --- | --- | --- |
| **Interaction** | **Negative Affectivity** | -0.31***  [-0.44, -0.17] | -0.13**  [-0.22, -0.05] | 0.02  [-0.07, 0.10] | 0.15 | 0.28 |
|  | **Detachment** | -0.34***  [-0.47, -0.20] | -0.08*  [-0.17, 0.00] | 0.01  [-0.10, 0.07] | 0.17 | 0.31 |
|  | **Psychoticism** | -0.22**  [-0.36, -0.07] | 0.05  [-0.14, 0.04] | -0.02  [-0.11, 0.08] | 0.07 | 0.26 |
|  | **Disinhibition** | -0.32***  [-0.47, -0.18] | -0.07  [-0.16, 0.02] | 0.03  [-.06, 0.12] | 0.11 | 0.27 |
|  | **p-Factor** | -0.41***  [-0.54, -0.27] | -0.09*  [-0.17, 0.00] | 0.01  [-0.07, 0.10] | 0.20 | 0.31 |
| **No Interaction** | **Negative Affectivity** | -0.29***  [-0.35, -0.22] | -0.13**  [-0.22, -0.05] | - | 0.15 | 0.28 |
|  | **Detachment** | -0.36***  [-0.42, -0.29] | -0.10*  [-0.18, -0.02] | - | 0.18 | 0.30 |
|  | **Psychoticism** | -0.23***  [-0.30, -0.17] | -0.08  [-0.17, 0.01] | - | 0.08 | 0.25 |
|  | **Disinhibition** | -0.28***  [-0.34, -0.21] | -0.10*  [-0.19, -0.02] | - | 0.12 | 0.26 |
|  | **p-Factor** | -0.39***  [-0.45, -0.32] | -0.09*  [-0.17, 0.00] | - | 0.20 | 0.31 |

*Note: AMPD = Alternative Model of Personality Disorders;* $\beta_{W}$ ***=*** *within-pair effect;* $\beta_{B}$ ***=*** *between-pair effect;* $\beta_{I}$ ***=*** *Interaction effect with zygosity; CI = Confidence Interval.*

** p < 0.05; ** p < 0.01; *** p < 0.001*
